# Mitochondrial Uncoupling and the Reprograming of Intermediary Metabolism in Leukemia Cells

**DOI:** 10.3389/fonc.2013.00067

**Published:** 2013-04-02

**Authors:** Juliana Vélez, Numsen Hail Jr., Marina Konopleva, Zhihong Zeng, Kensuke Kojima, Ismael Samudio, Michael Andreeff

**Affiliations:** ^1^Grupo de Terapia Celular y Molecular Laboratorio de Bioquimica, Pontificia Universidad JaverianaBogotá, Colombia; ^2^Section of Molecular Hematology and Therapy, The University of Texas MD Anderson Cancer CenterHouston, TX, USA; ^3^Department of Leukemia, The University of Texas MD Anderson Cancer CenterHouston, TX, USA

**Keywords:** Krebs cycle, mitochondrial uncoupling, electron transport, OXPHOS, apoptosis, anaplerosis, cataplerosis, microenvironment

## Abstract

Nearly 60 years ago Otto Warburg proposed, in a seminal publication, that an irreparable defect in the oxidative capacity of normal cells supported the switch to glycolysis for energy generation and the appearance of the malignant phenotype (Warburg, 1956). Curiously, this phenotype was also observed by Warburg in embryonic tissues, and recent research demonstrated that normal stem cells may indeed rely on aerobic glycolysis – fermenting pyruvate to lactate in the presence of ample oxygen – rather than on the complete oxidation of pyruvate in the Krebs cycle – to generate cellular energy (Folmes et al., 2012). However, it remains to be determined whether this phenotype is causative for neoplastic development, or rather the result of malignant transformation. In addition, in light of mounting evidence demonstrating that cancer cells can carry out electron transport and oxidative phosphorylation, although in some cases predominantly using electrons from non-glucose carbon sources (Bloch-Frankenthal et al., 1965), Warburg’s hypothesis needs to be revisited. Lastly, recent evidence suggests that the leukemia bone marrow microenvironment promotes the Warburg phenotype adding another layer of complexity to the study of metabolism in hematological malignancies. In this review we will discuss some of the evidence for alterations in the intermediary metabolism of leukemia cells and present evidence for a concept put forth decades ago by lipid biochemist Feodor Lynen, and acknowledged by Warburg himself, that cancer cell mitochondria uncouple ATP synthesis from electron transport and therefore depend on glycolysis to meet their energy demands (Lynen, 1951; Warburg, 1956).

## Oxidizing Carbon Sources Other than Glucose

**Figure 1:**
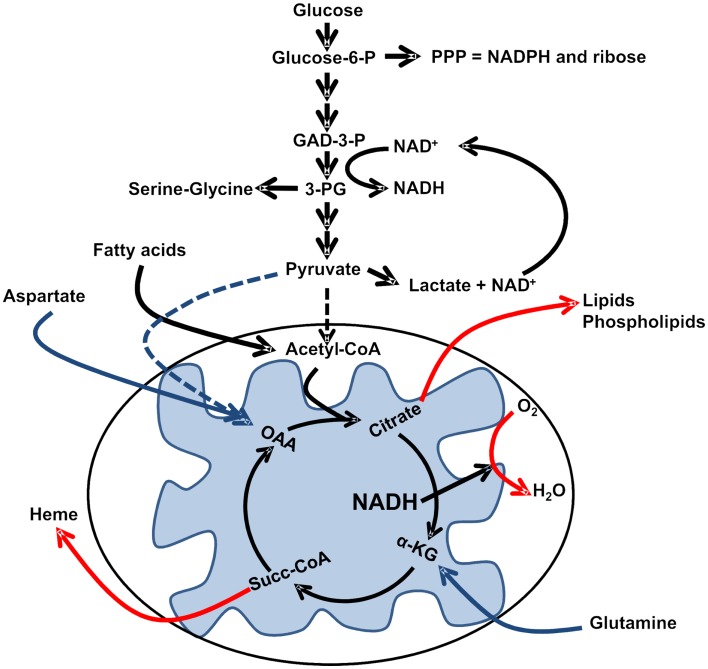
**Reprogramed pathways of intermediary metabolism**. Incomplete oxidation of glucose carbon skeletons provides intermediaries for biosynthesis, while fermentation of pyruvate to lactate regenerates NAD^+^ for the glycolytic conversion of glyceraldehydes-3-phosphate (GAD-3-P) to 1,3-bisphosphoglycerate and subsequent formation of 3-phosphoglycerate (3-PG). In order to maintain an adequate supply of biosynthetic intermediates in the absence of pyruvate-derived oxaloacetate (OAA), Krebs cycle activity relies on glutamine anaplerosis to regenerate alpha-ketoglutarate (α-KG; and potentially aspartate anaplerosis). Regeneration of cataplerotic citrate also relies on FAO-derived acetyl-CoA. Please refer to the text for additional details.

It has been shown that leukemia cells generate significant amounts of lactate even in the presence of adequate amounts of oxygen (Samudio et al., 2008, 2010) recapitulating Warburg’s observations in Ehrlich’s ascites tumor cells (Warburg et al., 1924). However, in contrast to what Warburg hypothesized, it is evident that leukemia cells have the ability to reduce molecular oxygen utilizing electrons from carbon sources other than pyruvate (Samudio et al., 2008, 2010). This finding is intriguing in light of the finding that in other tumor types, like glioblastoma, the anaplerotic (see Table 1) entry of glutamine-derived glutamate fuels the Krebs cycle and oxidative phosphorylation (OXPHOS) (DeBerardinis et al., 2007), suggesting that shunting pyruvate away from mitochondrial oxidation may be an acquired trait of different types of cancer. In the case of leukemia cells, recent evidence suggests that fatty acid-derived acetyl-CoA fuels Krebs cycle activity and the molecular reduction of oxygen (Samudio et al., 2010). Unpublished observations from our group suggest that glutaminolysis is essential to maintain oxygen consumption in these cells, supporting the notion that leukemia cell mitochondria are less prone to oxidize pyruvate (Figure 1). The question arises therefore, what could be the selective advantage of this metabolic shift?

**Table 1:** **Glossary of terms**.

Anaplerosis	Replenishing of Krebs cycle intermediates that have been used for anabolic purposes
Cataplerosis	Utilization of Krebs cycle intermediates for anabolic purposes
Amphibolic	A characteristic of the Krebs cycle that allows it to participate in catabolism of carbon skeletons, while at the same time, or at distinct moments, providing anabolic intermediates
Allosterism	Enzyme regulation (positive or negative) in a site distinct from the catalytic site
OXPHOS	Oxidative phosphorylation. The production of ATP in response to an electrochemical proton gradient generated during electron transport in the mitochondria
Mitochondrial uncoupling	The uncoupling of electron transport from ATP synthesis
FAO	Fatty acid oxidation; produces large amounts of reducing intermediates FADH_2_ and NADH, and promotes mitochondrial uncoupling; odd-numbered fatty acids can provide anaplerotic succinyl-CoA
Glycolysis	Catabolism of glucose into pyruvate; mostly oxygen independent, particularly when NAD+ is regenerated by fermentation of pyruvate to lactate
Glutaminolysis	Conversion of glutamine into glutamate; glutamate can be oxidatively or non-oxidatively converted to anaplerotic α-ke to glutarate
ROS	Reactive oxygen species; produced by NADPH oxidases, and of particular importance, produced by OXPHOS complex I and complex III; low levels appear to be required for normal cell functioning, whereas levels above particular thresholds are overtly cytotoxic

First, while complete oxidation of glucose in the Krebs cycle yields more ATP per mole of glucose than does glycolysis, it fails to safeguard carbon skeletons for anabolic reactions. For instance, pyruvate transamination produces alanine; alanine accumulation in turn allosterically inhibits pyruvate kinase, and results in accumulation of phosphoenolpyruvate which via equilibrium reactions is converted to 3-phosphoglycerate which serves as a precursor to serine and glycine (reviewed in DeBerardinis, 2011). Phosphoenolpyruvate is also an inhibitor of phosphofructokinase, which would shunt fructose-6-phosphate to glucose-6-phosphate that can now enter the pentose phosphate shunt to produce NADPH for biosynthesis and ribose-5-phosphate for nucleotide biosynthesis (Berg et al., 2002). Thus, non-oxidative metabolism of glucose carbon skeletons is essential for safeguarding biosynthetic capacity in rapidly proliferating cells.

Second, while the activity of the Krebs cycle has indeed a major influence in the regulation of OXPHOS-mediated ATP generation in transformed leukocytes, as these cells proliferate uncontrollably, OXPHOS is also likely to be affected by *de novo* pyrimidine synthesis. *De novo* pyrimidine synthesis is indispensable in rapidly proliferating cells in order to provide the heterocyclic aromatic precursors required for DNA, RNA, phospholipid, and glycoprotein formation (Evans and Guy, 2004; Hail et al., 2010b). This is especially true *in vivo* given the fact that the liver keeps the circulating levels of pyrimidines relatively low, thus limiting the role of pyrimidine salvage in the biochemical processes linked with cell proliferation (Traut, 1994).

**Figure 2:**
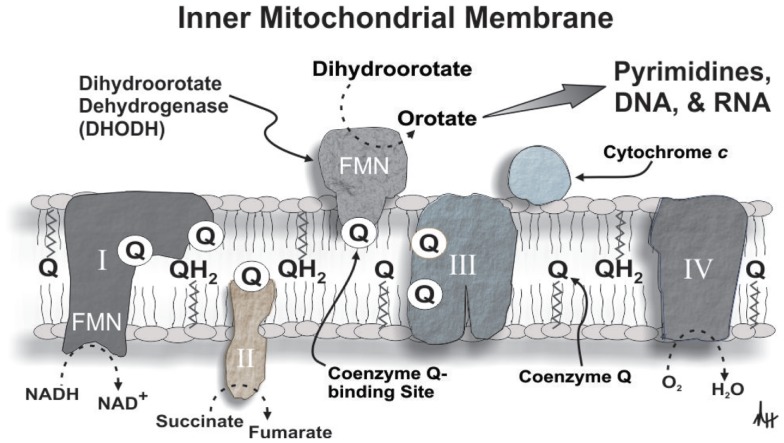
**A diagrammatic depiction of DHODH in the inner mitochondrial membrane illustrating its role in mitochondrial bioenergetics and *de novo* pyrimidine synthesis**. Please refer to the text for additional details (abbreviations: I, complex I; II, complex II; III, complex III; IV, complex IV; FMN, flavin mononucleotide).

Specifically, the oxidation of dihydroorotate via the activity of dihydroorotate dehydrogenase (DHODH, EC 1.3.99.11, the rate-limiting enzyme for the *de novo* pathway of pyrimidine synthesis) provides electrons for OXPHOS in a Krebs cycle- and glucose-independent manner thereby supporting mitochondrial bioenergetics and the proliferative capability of various cell types (Löffler, 1989), including hematopoietic cells (Xu et al., 1996; Rückemann et al., 1998; Sawamukai et al., 2007; Ringshausen et al., 2008). Coenzyme Q functions as the proximal electron acceptor for the oxidation of dihydroorotate to orotate by DHODH, and cytochrome *c* oxidase serves as the ultimate electron acceptor for this reaction. In this scenario, dihydroorotate functions as a reducing equivalent like NADH or succinate to modulate mitochondrial OXPHOS (Hail et al., 2010a) (Figure 2). In fact, the activity of DHODH is believed to be a major contributor to mitochondrial oxygen consumption in leukemia cells (Beuneu et al., 2000). Consequently, if DHODH and *de novo* pyrimidine biosynthesis are constitutively active in transformed hematopoietic cell this would not only affect their rate of proliferation (Shawver et al., 1997; Rückemann et al., 1998), but also their endogenous mitochondrial reactive oxygen species (ROS) production (Forman and Kennedy, 1975; Lakaschus et al., 1991; Lenaz, 2001). The very nature of this paired metabolic activity could serve as a feed-forward mechanism for leukemogenesis since ROS play an integral role in mutagenesis and oncogenic signaling (Hail and Lotan, 2009). Furthermore, DHODH activity in response to cell proliferation can function under a seemingly wide (i.e., ≥0.13%) range of oxygen tension, suggesting that aerobic conditions bordering on moderate hypoxia are theoretically sufficient to support OXPHOS and *de novo* pyrimidine synthesis (Löffler, 1989; Amellem et al., 1994).

Third, although OXPHOS is metabolically more efficient than glycolysis in terms of ATP generation, glycolysis occurs in the cytosol which typically represents >70% of the cell volume (Luby-Phelps, 2000) and thus has the potential of matching net ATP contributions from OXPHOS which occurs in the smaller volume of the mitochondrial matrix and inner membrane. In addition, OXPHOS depends on adequate amounts of oxygen and may not be sustainable under high rates of electron transport in rapidly dividing cells. Furthermore, oxygen levels are markedly reduced in the leukemic bone marrow niche (Benito et al., 2011). In discussing this latter point, it is important to consider that although glycolysis depends on the availability of NAD^+^ for the reaction catalyzed by glyceraldehyde-3-phosphate dehydrogenase, limiting the availability of oxygen – the canonical final acceptor of electrons from NADH – may not be critical as this oxidized cofactor can be readily formed by the fermentation of pyruvate to lactate. As such, depending on glycolysis instead of OXPHOS becomes a distinct advantage for proliferating leukemia cells in the hypoxic bone marrow niche.

Fourth, reduced dependence on OXPHOS for ATP generation favors the use of Krebs cycle intermediates for biosynthetic reactions. For instance, citrate cataplerosis from the Krebs cycle provides cytosolic acetyl-CoA for *de novo* fatty acid synthesis (FAS), and succinyl-CoA cataplerosis from the cycle provides carbon skeletons for the synthesis of heme groups (Berg et al., 2002) (Figure 1). These cataplerotic reactions of the Krebs cycle not only provide intermediates for biosynthesis, but when followed by anaplerotic reactions to regenerate oxaloacetate, also reduce the amount of NADH generated in the cycle. One should consider that citrate cataplerosis followed by α-ketoglutarate anaplerosis via glutaminolysis produces one less NADH than if citrate continued in the cycle; similarly, succinyl-CoA cataplerosis followed by oxaloacetate anaplerosis via transamination of aspartate or carboxylation of pyruvate produces one less NADH and one less FADH2 than if succinyl-CoA continued in the cycle. Taken together, it is intriguing to postulate that a “leaky” or truncated Krebs cycle can bypass in large part the requirement for sustained oxygen levels while at the same time providing critical intermediates for biosynthetic reactions.

Lastly, seminal work by Bonnet et al. (2007) demonstrated that in solid tumor cell lines the mitochondrial oxidation of pyruvate – induced pharmacologically by dichloroacetate (DCA) – promotes ROS generation and cell death, suggesting that oxidizing other carbon sources different from glucose may *ipso facto* be favorable in terms of cell survival. While there is no experimental evidence of a similar toxic effect of pyruvate oxidation in leukemia cells, it is important to consider the possibility that leukemia cells may also forgo pyruvate oxidation to maintain cell viability.

## Regulatory Adaptations

Leukemia cells must have acquired mechanisms to bypass the inhibitory effect of ATP accumulation on glycolysis, particularly at the point of phosphofructokinase 1 (PFK-1), the canonical regulator of glycolysis that carries out the conversion of fructose-6-phosphate into fructose-1,6-bisphosphate. Recent evidence suggests that in lymphoma (Nb2-11) and leukemia (TF-1) cell lines, PFK-1 has been post-translationally modified via a proteinase K-type of mechanism resulting in a shorter polypeptide which maintains catalytic activity, but is missing the allosteric sites required for inhibition by citrate, ATP, and phosphoenolpyruvate (Smerc et al., 2011). Intriguingly, the truncated enzyme is super-activated by fructose-2,6-bisphosphate (Smerc et al., 2011). Although similar modifications have not been reported in primary leukemia samples, it is intriguing to speculate that bypassing inhibitory effects of intracellular ATP accumulation could generate a critical growth advantage of leukemia vs. normal progenitors.

In addition to resisting the inhibitory effects of ATP accumulation on glycolysis, leukemia cells also use mitochondria-derived ATP to accelerate the rate of glucose activation and hence glycolysis. Over two decades ago, Arany et al. (1988) reported that the glycolytic enzyme hexokinase promoted oxygen consumption in leukemia cells when it associated with the outer mitochondrial membrane, suggesting perhaps a novel link between glycolysis and electron transport. More recent evidence (reviewed in Mathupala et al., 2010) indeed supports the notion that a particular isoform of hexokinase (HK), HKII associates with mitochondria to attain direct access to OXPHOS generated ATP for the phosphorylation of glucose. Additionally, the kinase AKT – a known activator of glycolysis in leukemia cells – phosphorylates HKII and is thus promoting its localization to the outer mitochondrial membrane (Majewski et al., 2004a,b). Phosphorylated HKII associates with VDAC, the outer mitochondrial membrane anion channel that allows entry of most metabolites, but only permits egress of pro-apoptotic proteins when the intrinsic pathway of programed cell death is activated (reviewed in Shoshan-Barmatz et al., 2010; Shoshan-Barmatz and Ben-Hail, 2012). Since mitochondria-bound HKII inhibits apoptosis in leukemia and lymphoma cell lines (Chen et al., 2009), it is intriguing to speculate that HKII prevents cytochrome *c* release by blocking the channel space in VDAC (Abu-Hamad et al., 2008), although conflicting reports suggest that the antiapoptotic effect of HKII may be instead dependent on the presence and/or activity of Cyclophilin D and ANT (Chiara et al., 2008). Regardless of the precise mechanisms, it is clear that HKII critically links mitochondrial metabolism, glycolysis, and survival in leukemia cells.

## Mitochondrial Uncoupling: Lessons from the Microenvironment

In 1956, Otto Warburg acknowledged an alternative hypothesis put forth by Feodor Lynen suggesting that the increased dependence of cancer cells on glycolysis stems not from their inability to reduce oxygen, but rather from their inability to synthesize ATP in response to the mitochondrial proton gradient [ΔΨM (Warburg, 1956)]. Lynen’s hypothesis was based on his own observations (Lynen, 1951) and previous work by Ronzoni and Ehrenfest (1936) using the prototypical protonophore 2,4-dinitrophenol (2,4-DNP), which causes a “short circuit” in the electrochemical gradient that abolishes the mitochondrial synthesis of ATP, and decreases the entry of pyruvate into the Krebs cycle. Since Ronzoni and Ehrenfest (1936) observed that 2,4-DNP induced an increase in oxygen consumption, and at the same time an increase in pyruvate conversion to lactate, it must have been tempting to hypothesize (although they did not) that electrons for the molecular reduction of oxygen must be coming from carbon sources different from glucose. Recent work has supported this hypothesis by demonstrating that the abrogation of ATP synthesis in response to ΔΨM (“mitochondrial uncoupling”) results in a preferential oxidation of non-glucose carbon sources to maintain mitochondrial function and an increase in lactate generation under aerobic conditions (Samudio et al., 2008, 2010; Sheets et al., 2008).

In leukemia cells mitochondrial uncoupling has been reported to recapitulate the Warburg effect and support a shift toward fatty acid oxidation (FAO) (Samudio et al., 2008, 2010). On one hand, it is important to point out that FAO induces an uncoupling and/or thermogenic phenotype in various cell types (reviewed in Gambert and Ricquier, 2007); on the other hand it is also evident that increased FAO during mitochondrial uncoupling may require anaplerotic reactions – in particular pyruvate carboxylation, glutaminolysis, and aspartate transamination – to replenish oxaloacetate for efficient Krebs cycle use of fatty acid-derived acetyl-CoA (Goodwin and Taegtmeyer, 2000). Indeed glutaminolysis has been reported in lymphoma cell lines (Le et al., 2012), and unpublished observations from our group suggest that glutamine supports oxygen consumption in leukemia cells. Taken together, the above data support the concept – and indirectly, Lynen’s hypothesis – that the Warburg effect could be the result of glutaminolysis-dependent FAO.

## Mitochondrial Uncoupling and Cell Survival: The Role of Bcl-2

In leukemia cells mitochondrial uncoupling promotes resistance to intrinsic apoptosis via, in part, antagonism of Bax/Bak oligomerization (Samudio et al., 2010). Uncoupling and chemoresistance are exacerbated in leukemia cells cultured on mesenchymal stromal feeder layers (Samudio et al., 2008), and previous reports indicate that stromal coculture upregulates Bcl-2 and Bcl-xL in leukemia cells (Konopleva et al., 2002) suggesting that the leukemia microenvironment promotes chemoresistance via, in part, by potentiation of the antiapoptotic function and/or expression of Bcl-2 family members. In addition, since mitochondrial uncoupling in leukemia cells is associated with a shift toward FAO and away from glucose oxidation (Samudio et al., 2010), it is tempting to speculate that the leukemia microenvironment also signals leukemia cells to spare glucose carbon skeletons for the generation of biomass (Samudio et al., 2009). Notably, pharmacologic inhibition of FAO was cytotoxic and chemosensitizing to leukemia cells cultured on bone marrow stromal feeder layers, promoting Bax/Bak oligomerization (Samudio et al., 2010). Most importantly, pharmacologic inhibition of FAO demonstrated a therapeutic benefit in combination with chemotherapy in mouse models of human leukemia (Samudio et al., 2010), suggesting that the shift toward FAO is a *bona fide* target for the treatment of hematological malignancies. Nevertheless, it is unclear whether FAO or electron transport *per se* antagonizes apoptosis in leukemia cells. In addition, the mechanisms by which FAO and/or electron transport regulate Bax/Bak oligomerization remain to be elucidated.

Recent work has suggested that Bcl-2 overexpression defines a subset of OXPHOS-dependent, ROS-low, quiescent leukemia stem cells (LSC), in which pharmacologic or genetic inhibition of Bcl-2 rapidly diminishes oxygen consumption capacity and increases ROS generation prior to the onset of apoptosis (Lagadinou et al., 2013). Although the precise mechanisms by which pharmacologic inhibition of Bcl-2 antagonize electron transport in LSC remain unclear, it is known that Bcl-2 displays an antioxidant function in mitochondria by facilitating the import of reduced glutathione into the mitochondrial matrix (Wilkins et al., 2012), and by directly reducing ROS generation (Low et al., 2010). In addition, Bcl-2 protects leukemic cells against mitochondrial uncoupling-induced apoptosis (Armstrong et al., 2001), and multiple other cell types against palmitate-induced apoptosis (de Pablo et al., 1999; Kui et al., 2009), suggesting that Bcl-2 may protect cells under metabolic overload of the electron transport chain. In light of the above, it is tempting to speculate that dependence on OXPHOS or increased rates of electron transport – such as observed in mitochondrial uncoupling – necessitates the accompaniment of Bcl-2 to safeguard mitochondrial integrity. It is of utmost importance to elucidate the precise molecular events that connect Bcl-2, apoptosis, and electron transport in leukemia cells in order to develop effective therapies to eradicate the elusive LSC.

## The Hypoxic Bone Marrow Niche and Metabolism: The Reverse Krebs Cycle

It is intriguing that in the bone marrow, although it constitutes a highly vascularized sinusoidal cavity, the oxygen concentration appears to be similar to that found in venous blood (i.e., 6%) (Fiegl et al., 2009) or even lower in the stem cell niche (i.e., 1%) (Benito et al., 2011), suggesting the notion that the bone marrow is *ipso facto* hypoxic, at least physiologically. However, if leukemia cell mitochondria are indeed uncoupled *in vivo*, the mechanism by which oxygen tension is reduced in the bone marrow may be directly related to the increased oxygen reducing capacity of leukemia cells. Still, this presents an interesting paradox: the reduced oxygen tension would eventually lead to an inhibition of Krebs cycle activity – via accumulation of NADH – and the reduced generation of ATP and biomass. Or will it? Intriguing recent research has suggested that under hypoxic conditions the Krebs cycle can actually go in reverse, starting from glutamine-derived α-ketoglutarate to form citrate via reductive carboxylation, to support the *de novo* synthesis of fatty acids (Wise et al., 2011; Metallo et al., 2012). Although no evidence of this effect has been reported in leukemia cells under hypoxia (Figure 2 speculates on the possibility of this event), it is tempting to consider that reductive carboxylation of α-ketoglutarate may rescue biomass generation from the Krebs cycle in leukemia cells under reduced oxygen tension, whether this resulted from cell autonomous consumption of available oxygen, or from yet unidentified anatomical and/or microenvironmental effects. Likewise, it is intriguing to consider that if reductive carboxylation of α-ketoglutarate is accompanied by increased conversion of NADH to NADPH (via NADH kinase), it may also represent a rescue mechanism against the inhibitory effects of NADH accumulation on the Krebs cycle under conditions of reduced oxygen partial pressure. Nevertheless, the reverse Krebs cycle faces some theoretical hurdles that would have to be addressed. First, it is unclear if sufficient amounts of α-ketoglutarate, ${\text{HCO}}_{3}^{-}$, and NADPH (reductive carboxylation of α-ketoglutarate requires NADPH via mitochondrial IDH2) are available in the mitochondrial matrix to promote the thermodynamically spontaneous formation of isocitrate. Second, it is kinetically improbable that isocitrate dehydrogenase, an enzyme that does not contain biotin, can catalyze carboxylation of α-ketoglutarate to form isocitrate. Perhaps a human ortholog of 2-oxoglutarate carboxylase (found in a variety of microorganisms; Reaction: ATP + 2-oxoglutarate + ${\text{HCO}}_{3}^{-}$ = ADP + phosphate + oxalosuccinate; EC 6.4.1.7) that can more readily catalyze carboxylation of α-ketoglutarate (2-oxoglutarate) into oxalosuccinate, which is then converted to isocitrate by IDH2, could account for the observed reversal of the Krebs cycle. However, whether this enzyme activity is expressed in leukemia cells, and whether leukemia cells can in fact generate isocitrate from – ketoglutarate, remains to be determined.

## Concluding Remarks: Warburg and Weinhouse Reconcile

A preponderance of evidence supports Warburg’s observation that cancer cells rely on glycolysis to meet their energy demands, although common, generalized defects in mitochondrial oxidative capacity have yet to be identified for the vast majority of cancers. In fact, more recent evidence has confirmed that cancer cells maintain the ability to oxidize pyruvate, albeit under conditions that were pharmacologically induced by DCA. Nevertheless, the concomitant ROS generation caused by this process is conceivably cytotoxic and, as such, could impinge on cell viability (Bonnet et al., 2007). At first, it may seem intriguing that Warburg’s hypothesis – that an irreparable damage to the oxygen reduction capacity of tumor cells promotes the origin of cancer – survived for nearly 50 years, especially as experimental evidence generated by contemporary biochemist Sydney Weinhouse demonstrated that tumor cells consumed oxygen at a rate similar, or higher than, their normal counterparts (reviewed in Weinhouse, 1972). Additionally, to biochemistry students familiar with the amphibolic importance of the Krebs cycle, and its apparent dependence on oxygen to accept electrons from NADH for it normal “clockwise” functioning, it must seem like an obvious conclusion that proliferating cells – tumor or normal – require an intact oxygen reduction capacity to generate important metabolic intermediates such as citrate, α-ketoglutarate, succinyl-CoA, and oxaloacetate. However, one must remember that the Krebs cycle was unknown to Warburg for nearly 10 years after his initial observations of respiratory impairment of cancer cells, when his former research assistant, Sir Hans Adolf Krebs, published two seminal papers that described the citric acid cycle (Krebs and Johnson, 1937; Krebs et al., 1938). Moreover, it then took several decades beyond that to fully understand the amphibolic nature of the Krebs cycle. In the end, Warburg’s imposing personality and his outstanding achievements ensured that his hypothesis survived – nearly unchallenged if not for Weinhouse – for almost 50 years.

Can the observations of Weinhouse and Warburg be reconciled? The evidence presented here suggests that, at least in leukemia cells, they can. On one hand, our recent data support Feodor Lynen’s hypothesis that increased lactate generation can be promoted under normal oxygen tension through mitochondrial uncoupling – induced by coculture with bone marrow mesenchymal stromal cells – in the absence of permanent, transmissible defects in oxygen consumption capacity. On the other hand, our observation that leukemia cells sustain molecular oxygen reduction rates utilizing electrons derived from FAO is in agreement with the work of Weinhouse utilizing transplantable hepatomas (Bloch-Frankenthal et al., 1965). Indeed, it would appear that Warburg and Weinhouse were looking at two sides of the same coin: aerobic glycolysis and FAO, which are elegantly connected by Feodor Lynen’s mitochondrial uncoupling theory.

While is important to point out that this review has focused predominantly in hematological malignancies, our data parallels recent observations in solid tumors where glutamine-dependent, glucose-independent Krebs cycle activity has been reported in glioblastoma and melanoma cells (Scott et al., 2011). In addition, our observation that mitochondrial uncoupling in leukemia cells is associated with increased resistance to apoptosis is consistent with the work of Derdak et al. (2008) who demonstrated a similar paradigm in colon cancer cells. As such, we hypothesize that our model may indeed be broadly applicable to other tumor types since there is presently insufficient evidence to suggest otherwise.

**Figure 3:**
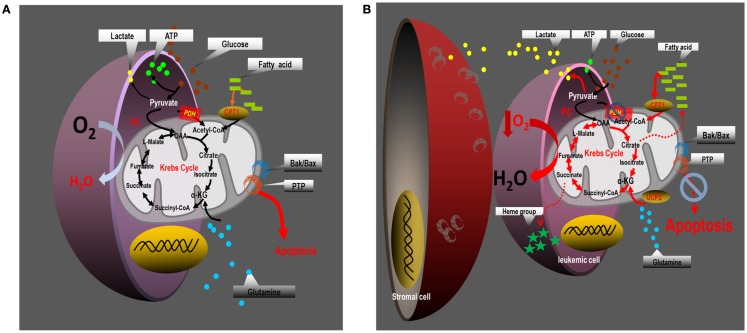
**Proposed paradigm for the reprograming of intermediary metabolism in leukemia cells**. **(A)** Leukemia cells cultured alone engage in aerobic glycolysis, and maintain an intact Krebs cycle which is capable of oxidizing pyruvate-derived acetyl-CoA, fatty acid-derived acetyl-CoA, and possibly glutamine carbon skeletons. Their energy demands are met through glycolysis and perhaps to some extent through mitochondrial OXPHOS, although mitochondria may display a proclivity for uncoupling electron transport from ATP synthesis. In monoculture, leukemia cells are most sensitive to apoptosis induction. **(B)** Coculture of leukemia cells with bone marrow-derived stromal cells promotes mitochondrial uncoupling. Under coculture conditions leukemia cells augment aerobic glycolysis and increase Krebs cycle activity in a manner dependent on anaplerotic reactions – in particular glutaminolysis, and to a lesser extent pyruvate carboxylation. The increased Krebs cycle activity does not oxidize pyruvate-derived acetyl-CoA, but instead metabolizes large quantities of fatty acid-derived acetyl-CoA to sustain increased rates of oxygen consumption, that may contribute to the generation of local hypoxic conditions. Mitochondrial uncoupling in leukemia cells is associated with increased expression of UCP-2, and increased resistance to apoptotic stimuli that induce Bax/Bak-dependent MOMP.

Based on analysis of the literature and our own data, we propose a paradigm (Figure 3) for mitochondrial uncoupling in hematological malignancies built on four salient foundations: (1) mitochondrial uncoupling promotes an adequate energy balance through aerobic glycolysis, and sustains biomass generation through cataplerotic reactions from the Krebs cycle; (2) mitochondrial uncoupling can orchestrate functional changes in mitochondria that accompany resistance to intrinsic apoptotic stimuli, mediated, in part, by modulation of the Bcl-2 rheostat; (3) mitochondrial uncoupling is regulated by, and regulates the bone marrow microenvironment, perhaps contributing to cellular hypoxia; and (4) mitochondrial uncoupling depends on anaplerotic reactions to regenerate oxaloacetate in the Krebs cycle to support fatty acid-dependent oxygen consumption. Whether or not this paradigm can be targeted for therapeutic benefit in patients with leukemia will largely depend on a thorough understanding of the limiting reactions that support the altered metabolic behavior of leukemia cells.

## Conflict of Interest Statement

The authors declare that the research was conducted in the absence of any commercial or financial relationships that could be construed as a potential conflict of interest.
